# Dr. Scudamore on Rheumatism, Rheumatic Neuralgia, and Tic Douloureux

**Published:** 1827-10

**Authors:** 


*A Treatise on the Nature and Cure of Rheumatism; with Observations on Rheumatic Neuralgia, and on Spasmodic Neuralgia, or Tic Douloureux.* By Charles Scudamore, M.D. F.R.S. &c. Octavo, pp. 589. Longman's, May, 1827.

The motto which Dr. Scudamore has chosen for his work, is taken from La Bruyere, and is very significant. Its meaning is this:—"I return to the public that which the public has lent me. I have taken from the public the materials of this work, and it is but just that I should make restitution." This is very honest, and we wish all medical men would pay their debts of honour to the public in this manner. True it is, that there are a very considerable number of authors who are *ultra-liberal* to the public; and who, never having borrowed any of the *rough materials*, are yet profuse in their presents of the *manufactured* article to that dear public which is so much the object of their generosity. The public may be divided into two portions—the professional and the non-professional. The former class are not the best patrons of monographs like the present volume, for we happen to know that not one work in ten of this description ever pays for the paper, and that, small as is the sale, more copies are bought up by the public than by the profession. In fact, it is almost inconceivable how little is known of the medical works that issue from the press (except through the medium of journals) among the great mass of medical society. Those who are in full practice have not *time* to read—those who are in little practice have not *money* to buy the books—and those who have sufficient time and money, want the *inclination* to wade through the products of the press. Still, with all these disadvantages, there must be something very seducing in authorship, to tempt hundreds annually to brave the dangers of criticism, and the still greater danger of neglect, in order to have the honour of giving birth to a book. Each aspiring spirit, in the medical art, is ready to exclaim—

> Tentanda via est quâ me quoque possum
> Tollere humo, victorque virum volitare per ora.

Dr. Scudamore is no respecter of persons. He has written for the benefit of Dives and Lazarus:—a guinea volume for the rich man with gout—and a sixteen shilling octavo for the poor man with rheumatism. In purchasing this last, the economist will save five shillings out of the fee, and have a world of advice for his money:—and as for DIVES, he can afford to have the book in his library, and the doctor himself in the drawing-room daily. If, therefore, the worthy author before us should not derive so rich a harvest from this last as from his first work, he will have the heart-felt satisfaction of reflecting that, "what is given to the poor is lent to the Lord," and will return with interest, in some shape or other.

Gout and rheumatism have been considered, even by one of the latest writers of the day,* as twin sisters; but surely this cannot be, for we find gout largely prevailing in the earlier ages of the world, when all was simplicity and temperance, while rheumatism is never mentioned:—and, on the other hand, now that the causes of gout have multiplied, we find rheumatism a far more prevailing complaint. It is evident that, if the disease was known to the ancients, it was confounded with gout, under the general name of arthritis, and no distinct notice was taken of the complaint till the beginning of the 17th century, when Ballonius left a posthumous work on the subject, describing it as a new disease. Then followed Sydenham, Hoffman, Stoll, Boerhaave, Sauvages, Musgrave, Huxham, Cullen, Barthez, and last, not *least*, Scudamore. We say, "not *least*," in respect to the size of the book, the "bulk" of which, Dr. S. seriously hopes, (p. xiii.) "will be found sufficiently *comprehensive*." We are quite convinced that Dr. Scudamore may make himself easy on that point. No complaint, we may venture to predict, will ever be made that the Doctor's volume on rheumatism is too small! In truth, 600 pages on a single subject—and that not the most interesting one in the circle of medical science, will be thought *satis superque*, by most of the profession, as Dr. S. will probably find in the course of a few years.† Be that as it may, we shall endeavour to offer a comprehensive view of Dr. Scudamore's doctrines and practice in this disease.

Dissatisfied with the definitions of his predecessors, our author has constructed one of his own. It is this:—

"*Pain of a peculiar kind, usually attended with inflammatory action,*

---

* M. Goupil, "Exposition des Principes de la Nouvelle Doctrine Medicale."

† Although Dr. Scudamore's is the largest work which we possess on the single subject of rheumatism, it does not contain so much matter as the laborious and erudite compilation of Villeneuve, which occupies upwards of 200 pages of small and closely printed letter-press, in the 48th volume of the *Dict. des Sciences Medicales*. From that source Dr. S. has probably drawn some of his materials, though he modestly designates them as *loans* from the public, now at last repaid.

*affecting the white fibrous textures belonging to muscles and joints, such as tendons, aponeuroses, and ligaments; the synovial membranes of the bursæ and tendons; and nerves; occasioned by the influence of variable temperature, or by direct cold, or by moisture.*" 12.

Comprehensive as is the above definition, yet we think it too confined. Thus the pleura, and the non-reflected portion of the pericardium are excluded from being the seat of rheumatic inflammation, as not "belonging to muscles and joints," though we think that most practical men will have observed rheumatic inflammation in those, and in many other parts not included in the above definition. "Almost all authors," says Villeneuve, "agree that the *fibrous system*, dispersed, under various forms, throughout the whole of the animal frame, is the principal seat of rheumatism." The mucous and serous membranes have been considered by many of our most able physicians, as occasionally the seat of rheumatic inflammation, and we have little doubt of the fact.* But, as we never attached much importance to definitions, we shall pass on to other topics.

The species of rheumatism are two, the acute and chronic—the former divisible into the acute and subacute.

It is quite needless to go over the symptoms and the causes, predisposing and exciting, of rheumatism. The former are familiar to every practitioner, and all that we know of the etiology was known before Dr. Scudamore's work appeared. In respect to the *proximate* cause, (the stumbling-block of theories and theorists,) we shall leave it to our readers how far Dr. S. has removed the veil from that of rheumatism, in the following passage.

"It may be stated, that the predisposition to rheumatism consists in a deficiency of healthy tone in the textures, connected with joints and muscles, and in nerves, so as to be affected in this peculiar manner by the influence of variable temperature. If we lose sight of the humoral term 'rheumatism,' we shall come to the simple fact, that, in a condition of susceptibility, cold, or sudden reduction of temperature, makes a particular impression on the vessels and nerves near the surface, and produces a painful affection of certain textures, which is attended with more or less of inflammation; the phenomena of which are so far of a peculiar nature, that we may either consider the disease *specific*, inas-

---

* Indeed, Dr. S. himself acknowledges, page 19, that "the fibrous parts of the more important and delicate organs are not exempted from rheumatism." Again—"when the inflammatory diathesis is active, *serous membranes* may be attacked with inflammation; but I should consider such affections in the light of coincidence, or consequence, and not as examples of the primary action of rheumatism."

much as the symptoms differ in their constituent character from those produced by other inflammations; or, we may view the effect in the light of common inflammation, modified on the one hand by the nature of the exciting cause—the external one, Cold—and, on the other hand, by the influence of the particular species of textures which become affected." 49.

In the next page Dr. S. observes that—"it may be stated that rheumatism, in its true primary character, as being a diseased condition of certain textures, is not *so distinctly* a constitutional disease as gout, which requires a specific state of constitution."

This passage surely involves a contradiction; for if rheumatism be merely "a diseased condition of certain textures," or, in plain terms, a local disease, it cannot be a constitutional one. If, on the other hand, it be a constitutional affection, its being *more or less* distinctly so than gout or any other disease, does not bear on the question. The fact is, that Dr. Scudamore is sadly embarrassed in the attempt to account for the general fever in rheumatism *preceding* the local affection, and yet being *caused* by that local affection. But let him speak for himself.

"Dr. Johnson, the ingenious author of a work 'On the influence of the atmosphere, &c.' seems to object to my opinion, that the fever in acute rheumatism is symptomatic; but I confess myself not convinced by his arguments. Do we not see that general fever (pyrexia) is chiefly proportioned to the nature and extent of the parts affected? If a single bursa be the sole seat of disease, the constitution does not actively sympathize. General fever, in this case, does not take place; but it is the invariable attendant on an inflammation of ligamentous or tendinous structure, existing at the same time in different joints. Although it occasionally happen, in point of time, that pyrexia precedes the development of the local rheumatic inflammation, may we not consider that the constitution has taken such early alarm from a ready sympathy with the textures upon which the offending agent, Cold, has certainly made its impression, notwithstanding the characteristic symptoms do not become all at once developed? Yet the interval between the occurrence of the constitutional fever and the local signs of inflammation is never long; and the general fever, inasmuch as it is truly rheumatic, keeps pace entirely with the local disease." 52.

This is very lame reasoning—in short, it is a mere *petitio principii*. If this be allowed, we may next assert that the fever of variola is merely symptomatic of the local inflammation of the pustules which *are to come out* a few days afterwards! Whereas, it is evident that the eruptive fever is the natural consequence of an agent that has impressed its deleterious influence on the system at large, although, from its peculiar nature, a cutaneous eruption occurs at a certain period of the disease—and then the fever is kept up or aggravated by the local affection. We conceive that it is just the same with rheumatic fever. An external cause—say atmospheric vicissitudes—acts on the system at large, (for we maintain that the cause acts generally, not locally) and is followed, first, by constitutional disturbance, exhibiting the phenomena of fever; and, secondly, by a local affection, in the shape of acute rheumatism. This last is no more the cause of the original fever, (though it may afterwards keep it up) than the fever is the cause of the local inflammation.

We would recommend Dr. S. to search the records of naval and military hospitals, and if he can find a considerable number of instances, where the symptomatic fever of gun-shot wounds shewed itself a few days, or even hours, before the battles in which the wounds were received, then we shall give up our opposition to his theory. Or he may investigate the matter in his own neighbourhood. In consequence of the numerous new buildings going forward in the west end of the town, there are cases of fracture of the limbs and skull almost daily brought into the Middlesex and St. George's Hospitals. If Dr. S. can prove that, in a good proportion of these cases, the symptomatic fever shews itself a few days or hours before the men fall from the house-tops, we shall give up the point at once, and acknowledge that the fever which precedes the local inflammation in rheumatism is the *effect* of a cause which *follows*.*

This discussion is not a matter of mere curiosity. It will bear on the treatment, as will be afterwards shewn.

Dr. Scudamore dedicates a few pages to the diagnosis between gout and rheumatism, and his diagnostics are unobjectionable. In well-marked cases of each disease, the diagnosis is easy enough, and where the symptoms of gout and rheumatism amalgamate, the treatment will not be so different as to render a diagnosis of any material consequence.

> " There is one circumstance which happily distinguishes acute rheumatism from acute gout: that it is not a disease of periodical occurrence, and that it very commonly spares the patient for the rest of life, although its solitary attack may have been peculiarly severe." 63.

---

* Indeed the author himself, a few pages farther on, virtually gives up the doctrine. He says, " it is, however, in an analytical point of view *only*, that I assign to rheumatism more of a local than a constitutional character. In a true comprehensive pathology, no disease, not the most simple, can be pronounced so local as to be independent of the constitution."

We have seen so many internal affections, especially of the heart, follow an attack of acute rheumatism, that we are not sure whether we should prefer this last disease, with all its improbability of recurrence, to gout, with the chance of frequent visitations afterwards. Gout may generally be warded off by early and persevering temperance, with proper exercise; but the insidious diseases of the heart, which steal on after acute rheumatism, give little warning to the victim till it is too late!

*Prognosis.* This, of course, is favourable, so long as rheumatism keeps to external parts; "but danger, more or less imminent, presents itself when any vital organ becomes affected, whether by metastasis or otherwise; but most where there is metastasis; because, in this case, the fibrous textures of the heart, or of the brain, (surely Dr. S. does not mean the brain, but its envelopes) usually become the seat of the disease, and those of the heart more frequently than of the brain." If the lungs should become affected by inflammation, during acute rheumatism, Dr. S. would not consider the phlogosis as rheumatic, "for they do not possess the necessary texture for this specific affection." We should think they have nearly as much of the fibrous texture as the brain, granting, as we do, with Dr. Spurzheim, that this last organ is fibrous. But we are far from being convinced that diseases have such regular and invariable limits, in their seats, as to be exclusively confined to this or that texture of the body.

*Treatment.* The following proemium to the therapeutical division of the work, would answer just as well for any disease in Cullen's Nosology, as for rheumatism.

"In the investigation of every case, he should enquire whether the rheumatic diathesis be hereditary, or acquired: he should acquaint himself with the peculiarities of the individual constitution, and also the patient's particular state of general health at the time of being attacked with this disease. He should scrutinize very carefully the condition of the digestive organs.

"Upon these and other data, he makes his choice of particular remedies; and, in this department of his knowledge, an acquaintance with the principles of pharmaceutical chemistry comes to his aid, enabling him to direct the fit combination of medicines, which may be wholly different in their nature, and which may oppose or assist each other, according to the skill of the arrangement. I need not dwell upon the value of experience in regard to a knowledge of diseases, and the efficiency of remedies." 68.

*Bleeding.* This measure, Dr. S. confesses, is "not the agent in which we should place our confidence, as far as the local inflammation is concerned, for it generally disappoints our expectation of relieving the pain of the disease, unless as the pain and the local inflammation may be connected with the true inflammatory diathesis."

"In no way is a degeneracy into chronic symptoms so certainly introduced, as by that injudicious employment of general bleeding, which enfeebles the constitution, and still leaves the rheumatic disposition in great force: Nor does the articular inflammation itself yield to the use of general bleeding in the manner which we might expect." 70.

Dr. S. limits general bleeding to the patient of strong muscular fibre, of sanguineous temperament, full health. If such a person be seized with acute rheumatism, he may be bled "until the hardness and fulness (of the pulse) become reduced to a state of softness and moderation." Cautious and judicious as this rule may appear, we are convinced that it will lead the young and inexperienced practitioner astray. While local inflammation and pain continue, we cannot reduce the pulse to "softness and moderation," if we were to drain the last drop of blood from the body; and, therefore, we suggest that the rule is dangerous. In such cases as the above, we would be inclined to say, take one or two copious bleedings at the beginning, so as to lessen the general mass of blood; but, after that, be guided by the appearance of labour or disordered function in the great organs of the body, and only bleed when some of these are threatened. Dr. S. justly remarks on the difficulty of distinguishing nice shades of difference in the pulse, and quotes Dr. Philip's observations on this subject, in a way which leaves us in doubt whether he approves, disapproves, or laughs at, the nice discrimination of this eminent physician and physiologist. John Hunter observed, that accuracy in distinguishing pulses was inattainable by many, "for simple sensation in the minds of any two men are (is) seldom alike." He instances the late Dr. William Hunter as a striking example; "for though he was extremely accurate in most things, he could never feel that nice distinction in the pulse that many others did, and was ready to suspect more nicety of discrimination than can really be found." The buffy coat of the blood in acute rheumatism is now well known to be very fallacious—in fact, that it affords us no criterion for the repetition of blood-letting.

"A surer practical indication may be taken from the form of the coagulum and its firmness. When it is exceedingly cupped, and when the inferior part beneath the stratum of fibrine is very firm, it is a presumptive evidence that the heart and arteries are labouring under that morbid contractility which distinguishes the inflammatory diathesis. A hardness of the pulse always attends this state of the blood." 74.

If the heart, brain, lungs, diaphragm, or other internal organ of importance, become affected with inflammation, we must bleed, of course, till the organ is relieved. It is useless to go over the authorities which are cited by Dr. S. in support of general bleeding in rheumatism. Would to God that each author would state the result of his own experience in remedies, without swelling his book with excerptæ from all other authors, from the flood downwards!

*Emetics* are lauded by Dr. Scudamore, as excellent remedies at the commencement of the disease. It is more than we can say for them—but Dr. S. is paramount authority, on this point.

*Purgatives.* It is curious that the only objection which Dr. S. urges against purgative medicines, in rheumatism, is—"the pain and difficulty attendant on frequent change of position." If Dr. S. ever treated himself to an emetic, he would know that the changes of position and action of muscles, consequent on vomiting, are at least equal to, if not greater, than those attendant on purgation.

"I have been much satisfied with the effects of a draught composed of the carbonate of magnesia, carbonate of potash, sulphate of magnesia in small doses, tartarised antimony, lemon juice in fit proportion to neutralize the carbonate of potash, and the acetum colchici, with some agreeable distilled water and syrup. The draught may be taken in effervescence, or otherwise. The addition of the tartar emetic is exceedingly valuable; for my increasing experience with this medicine convinces me that it is one of the most useful remedies which we can employ for the removal of inflammatory action; and in proportion as we employ it with judgment, so do we diminish the necessity of using the lancet. Its influence is much more permanent than that of digitalis; which, although on many occasions to be regarded as a most valuable auxiliary in the treatment of inflammation, is yet liable to the objection of its restraining action, rather than subduing inflammation, and masking, rather than curing the disease." 92.

In concurrence with this draught our author prescribes calomel with the compound extract of colocynth at night—the former, from one grain to five, continuing the mercurial, in urgent cases of acute rheumatism, till the mouth becomes slightly affected. It is usually expedient to add to the pills above-mentioned, a portion of opium, extract of poppy, or hyosciamus.

"In regard to the freedom, and continuance of this treatment, we shall inform ourselves, in great measure, by a regular observation of the nature of the excretions, alvine and urinary; for, while the fæces are unnaturally dark, and the urine is dense, of a deep colour, turbid, or even depositing lateritious or pink sediment, the fluid portion being clear, it is incumbent upon us to make daily employment of purgative medicine. When the excretions acquire a natural appearance, the acute symptoms of inflammation usually subside, and then our active treatment must be exchanged for the occasional use of a sufficient quantity of an aperient for the regulation of the bowels; at the same time, taking advantage of the absence of fever, to introduce the trial of tonic medicine, and restorative diet." 96.

If remissions of the fever be sufficiently distinct, the sulphate of quinine, or other form of bark, is to be given.

Under the head of "Tartar Emetic," our author introduces the opinions and practice of the late lamented Laennec, as grounded on the new Italian doctrine. But with this practice our readers are sufficiently acquainted. The following formula, combining the anodyne with the antimonial remedy, we copy from page 106 of the present work.

"℞. Potassæ Carbonat. gr. cviij.

Succ. citric (recentis), ℥ij.

Mist. Camphoræ, ℥iijss.

Liquoris opii sedativ. ʒiss. ad ʒij.

Syrupi tolutan. ℥ss.

Antimon. tartarisat. gr. i. ad gr. ij.

M.—Fiat Mistura." 106.

One, two, or three table-spoonfuls of the above mixture are to be given every hour or two till pain is relieved. Dr. S. also recommends the acetate of morphine, (from a quarter of a grain to a grain) in a saline draught, with camphor mixture, and a small portion of hydrocyanic acid. The following formula is recommended when the inflammatory diathesis does not prevail very strongly, and where general rheumatic pain and consequent irritation predominate.

"℞. Liquoris Ammon. Acetat. ℥ss.

Vini Colchici, ♏xx. ad ʒss.

Syrupi papaveris, ʒj.

Mist. Camphoræ, ℥j.—M. Fiat haustus sexta vel octava quaque hora sumendus." 107.

Where colchicum is found to disagree, the Dover's powder may be substituted, in a saline draught at bed-time, or oftener if necessary.

In respect to Peruvian bark, as given in considerable doses throughout the fever of rheumatism, by Haygarth and others, Dr. S. does not speak favourably from his own experience.

Indeed, it is seldom now employed till remissions appear—and then the sulphate of quinine will generally be found superior.

*Local Treatment.* Considering the tendency which the rheumatic inflammation has to migrate, Dr. S. does not place much dependence on local applications—with the exception of leeches. "But, unless some one part is severely painful, I should avoid the inconvenience of this remedy; and, if general bleeding be not required, I am satisfied to place my confidence in the internal means of treatment." When the general fever has subsided, and the remaining rheumatic inflammation possesses more of a distinct local character, then Dr. S. recommends a lotion "composed of *two* parts of alcohol and *one* of mistura camphoræ, applied tepid, by means of several layers of linen, and over them a piece of oil silk." If there be no mistake in the above lotion, we must say that we should reverse the proportions of the ingredients—or rather, we should put one part of alcohol to three or four parts of the camphor mixture, both on account of economy, and being sufficiently evaporating, without being too stimulant.

In convalescence, Dr. S. recommends the quinine in well acidulated infusion of roses, with tincture of cinchona, to recruit the strength.

*Metastasis.* This is an important feature in acute rheumatism. Dr. S. says it is not of frequent, "but it is a possible occurrence." We think the expression might have been stronger. Our own observations teach us that the metastasis is much more frequently insidious, and consequently more liable to be overlooked, than is imagined. For many years past we have paid considerable attention to diseases of the heart; and, on minute inquiry, we have found that, in the majority of cases, there had been one or more attacks of acute or sub-acute rheumatism previously. There may be no direct metastasis at the time the acute rheumatism occurs—but the disease of the heart often steals on afterwards, without any other ostensible cause than the rheumatic diathesis. Be that as it may, the metastasis occasionally takes place in an obvious and unequivocal shape.

"There is not, probably, a more dangerous form of disease, than a sudden seizure of the heart during the inflammatory state of the system in acute rheumatism.

"The chief symptoms of this alarming malady are, a hard and rapid pulse, rather small than full, and sometimes attended with irregularity; the breathing hurried and anxious; palpitation of the heart, with occasional pain in its region; some cough; a distressed countenance; beating of the carotids; the highest state of nervous irritability. The patient lies on his back, a little inclined to the left side, with his head raised, and dreads the least movement of the body; suffering at the same time great agitation of mind, and a restless desire of improving his position." 123.

Our author goes on to present an analytical digest of what has been written on the subject of rheumatic metastasis to the heart, beginning with Sir David Dundas's paper in the Medico-Chirurgical Transactions, and noticing the various accounts published by Baillie, Wells, Pitcairn, Odier, Corvisart, Burns, Forbes, Johnson, and particularly Laennec. In respect to the cause of this melancholy metastasis, our author may be said to be almost silent. For our own parts, we have had such direct evidence of the injurious effects of ultra-depletion, violent purgation, and warm-baths, in acute rheumatism, that we have no hesitation in placing to the account of these measures, many of the instances of metastasis which have occurred, and do occur. One of the most formidable metastases which we have witnessed, occurred, early in the present year, in the person of a strong man who, in his first attack, after two or three profuse venesections, was put into a warm-bath, from whence he emerged with great relief to the pain and inflammation in the joints, but with the supervention, in a few hours, of raging delirium, and furious action of the heart. The cerebral irritation, or perhaps inflammation, required numerous leeches and ice to the head—sinapisms—and brisk purgation. The head was relieved; but the action of the heart continued violent and extremely irregular for many weeks, although the rheumatism returned to the joints, and harrassed the patient for a long time. There is still considerable functional derangement of the central organ of the circulation, and we apprehend that enlargement of the ventricular parietes is gradually taking place. We agree with Dr. Scudamore in the following passage.

"The state of the digestive organs, and of the functions of the liver especially, will require particular attention, as having a very material reflected influence on the disease of the heart. This fact is so remarkable, that, in some instances, the almost only useful treatment will be found to consist in those means which rectify the action of the liver, and the condition of the digestive viscera in general.

"Regimen and moral management comprise a very important branch of our method of cure. An abstemious diet, almost confined to milk and vegetables, or one of medium nutrition, between the extreme plan and the ordinary use of animal food, should be directed, according to the character of the symptoms, and to the particular constitution of the patient. We should endeavour to support strength, and restrain action. Active exercise should be avoided, and airing in a carriage, open or closed, according to the season of the year, will be the most advisable mode of exercise." 145.

On the other metastases of rheumatism, as to the brain, diaphragm, eye, &c. Dr. Scudamore makes many judicious observations, but none such as to require particular notice in this place. Neither shall we stop to review the section on sub-acute rheumatism, but proceed to some cases in which metastasis occurred, and which are the most interesting in the volume.

*Case* 1. A gentleman, of irritable constitution, a bon vivant, and accustomed to great exercise, had enjoyed good health till the age of 48, when he was seized with acute rheumatism, (in the spring of 1823) by which he was left weak, and affected with chronic symptoms. At the end of 18 months, he became seriously indisposed with palpitation and nervous irritability in the highest degree.

"Of the exquisitely sensitive state of the patient's mind, I am unable to give an adequate description. If he attempted to take rest in bed, he was soon disturbed in his sleep by horrid dreams, and awoke suddenly with the most immediate dread of dying. He would obtain some repose afterwards in an easy chair, reclining a little, with the legs supported." 247.

His brother had died of disease of the heart, and this circumstance did not add to the tranquillity of his mind. He had sense of weight, but not of pain, in the region of the heart, with frequent pains in the middle of the arms—pulse irregular, and 100—tongue creamy white—copious colourless sediment in the urine—clay-coloured motions—bloated countenance—great fulness of the abdomen, especially of the right hypochondrium. His habits of regimen were intemperate—he paid no attention to his bowels—hated medicine—and took much horse exercise. Dr. S. was not then acquainted with auscultation, but concluded that the cardiac symptoms were owing to "engorgement of the vessels of the liver, which was also torpid in its secreting functions." Dr. S. prescribed pil. hyd. gr. iij. ex. col. comp. gr. iv. lactucar. gr. iij. every night, and a light senna draught the succeeding morning. In the day, two draughts, (saline) with digitalis and hydrocyanic acid. This methodus medendi, added to the cheering hopes thrown into the scale by the Doctor, soon produced a wonderful amendment, and in six weeks the urgent symptoms had ceased. Brighton air and sulphate of quinine completed the cure.

It required no stethoscope, nor, indeed, any very critical acumen, to determine, in such a case as the above, that the disordered action of the heart, and all the nervous symptoms, were owing to repletion and bad secretions.

*Case* 2. This case is headed "*Metastasis of Disease to the Heart.*" A gentleman, aged 47, had suffered twice from acute rheumatism, with an interval of 18 years. The second attack preceded the cardiac symptoms about three months. Chronic rheumatism was left after this last acute attack, affecting the synovial membranes, the ligaments, and tendinous structure. These chronic symptoms continued for a considerable time after the invasion of the cardiac disease. "Certainly there was no *abrupt* cessation of the rheumatic disease, on the supervention of that of the heart, which could possibly be considered in the light of metastasis." Then why, we ask Dr. S. does he head the case "Metastasis of Disease to the Heart?" Here is evidently a contradiction of terms. But it is of little consequence whether we call it a metastasis, or an extension of disease from exterior structures to internal organs, provided we know the fact, that there is a connexion between the one disease and the other.

The disease of the heart, in this case, was tedious in its course. The limbs finally became anasarcous, and death closed the scene. The heart was found nearly double its natural size—the auriculo-ventricular valves greatly ossified—serous effusion in the pericardium and thorax.

*Case* 3. The heading of the last case might well be transferred to this, without any reservation in the body of the case. A youth of seventeen was attacked with acute rheumatism, characterized by sudden transference from one part to another. "Suddenly, and with an abatement, but not cessation of the rheumatic inflammation of the limbs, the patient was seized with symptoms of pericarditis." Notwithstanding the most active treatment, the patient was cut off in three days.

Upon examination, the fibrous layer of the pericardium was found partially adhering to the heart, by recently-formed portions of fibrine; and the bag of the pericardium contained a few ounces of turbid serum.

*Case* 4. This case, on the other hand, has little connexion with the subject under consideration, as it is evidently one of idiopathic aneurism of the heart and aorta.

A gentleman, aged 39 years, had fatigued himself with exercise on a very hot day, and then went into a cold cellar. He was immediately taken ill, and lay twelve hours in a dangerous state—countenance cadaverously pale, lips and nails blue, and requiring strong stimulants "to avert fainting and keep on life." It was found that the intestines were greatly loaded, and by purgation, alteratives, and strict diet, he partially recovered. On taking quick exercise, however, he was subject to hurried respiration. In the autumn of the same year, after exercise, he was suddenly seized with syncope, and appeared to be dying. He was recovered by cordials, but these kinds of attack frequently returned afterwards. At the end of the following year, he was attacked with sub-acute rheumatism, chiefly affecting the muscles of the arm and chest. The cardiac symptoms became worse. Dr. S. found him with dark and unhealthy complexion—pulse remarkably dilated—respiration uncomfortable —indescribable sensations about the heart—palpitation and breathlessness on going up stairs, or on being mentally excited —various nervous symptoms, and great debility. The bowels and biliary secretion were carefully regulated, and the carbonate of iron was given. By these means his health was improved, and he could take a good deal of walking exercise without difficulty. But, in two months after this, the scene suddenly changed. He went to bed one night, "with very comfortable feelings," and slept calmly. He awoke in a few hours without agitation, turned on his right side, suddenly breathed with noise and difficulty, but not with stertor, and, in less than two minutes, expired.

The following were the appearances in the heart.

"It was very considerably increased in volume; there were firm and universal adhesions between the corresponding surfaces of the pericardium; the right auricle and ventricle were enlarged to twice their natural capacities; the edges of the tricuspid valve diseased; the left auricle and ventricle greatly enlarged, the former to twice, the latter to more than twice, its natural dimensions; edges of the mitral valve diseased; semilunar valves of the aorta thickened, with appearances of incipient ossification; this artery was very much enlarged, and its muscular coat was ruptured." 260.

The above case shews the insidious nature and deceptive phenomena of diseases of the heart. Auscultation would certainly have shewn the existence of the cardiac aneurism, and would have led the medical practitioner to have put patient and friends on their guard respecting the ultimate result. Would carbonate of iron have been exhibited, had the real nature of the disease been known? It is true that tonics and stimulants will give (sometimes at least) temporary relief to the nervous affections dependent on diseases of the heart; but we believe they generally accelerate the fatal result. Hypertrophia of the heart, or of any other organ, is but another name for *inordinate nutrition*, or morbid growth of the part. Is this likely to be checked or remedied by tonics or stimulants? In treating diseases of the heart, in particular, the physician ought to look to the nature and tendency of the organic lesion rather than to the morbid feelings of the patient. But, alas! he is too often compelled to attend to, and obviate these temporary and distressing phenomena, while the fatal *cause* is neglected!

*Case* 5. *Metastasis to the Brain.* A young lady, aged 15, of delicate constitution, had been labouring under acute rheumatism for a fortnight, in the upper and lower extremities. She complained suddenly one morning of severe pains in the head, the rheumatism still continuing in the limbs, without abatement. In the afternoon, Dr. S. found the patient in a comatose state. When roused, she observed that her limbs still ached, but her head was most in pain. The pulse was 130, and *small—countenance pale*—pupils greatly *contracted*—she appeared to be sinking. Leeches to the temples and forehead —blister to the neck—sinapisms to the feet. She died before midnight.

On dissection, considerable serous effusion was found between the arachnoid and pia mater, the vessels of the latter appearing turgid.

*Remarks.* We think it will be granted that, in the above case the symptoms indicated effusion rather than inflammation, the latter having passed. We therefore question the propriety of leeching under such circumstances. Had there been flushed face, ferretty eyes, delirium, and quick, hard pulse, the sanguineous evacuation would have been indicated unquestionably; but these phenomena did not obtain in the above case.

Dr. S. occupies the next 22 pages with cases of rheumatic metastasis, from the work of the younger Andral, recently published. But, as we shall review M. Andral's book, we need not stop to notice any of the cases on the present occasion.

*Case* 6. At page 289, there is a case given to the author by Sir Astley Cooper, and Dr. Young of Lambeth, some particulars of which we shall here record.

A gentleman, 60 years of age, stout and corpulent, of irritable constitution, and rather a free liver, not subject to gout and rheumatism, had suffered two years from stone in the bladder. Such was the morbid sensibility of the parts, that the introduction of a bougie generally created much pain, as did the passage of the urine. It was agreed that lithotomy should be performed; but a few days before the proposed operation, (the patient's mind being extremely anxious, and strongly impressed with the idea that it would prove fatal,) he was seized with rigors, head-ache, pain in the chest, and cough. The pulse was 96 and full, tongue white, skin hot and dry. There was also pain in the ankle of the left foot, but no external inflammation. The next night was passed in a restless manner—the pain had quitted the ankle, and was fixed in the knee, being very excruciating, and the part exceedingly sensible to the touch. The surface was shining—there was strong fever. On the third day, all the symptoms continued. On the fourth, the pain of the knee had become intense, with slight redness of the skin, pulse 120, general fever increased, abdomen tender. On the fifth day, (that fixed on for the operation,) all the symptoms were exasperated—vomiting supervened—the intellectual functions were deranged, and, after lingering a day or two longer, he died.

On examination of the body, the only morbid appearances were in the bladder and knee. The coats of the former were thickened, the inner surface highly vascular, the calculus, studded with small crystals, lay imbedded in mucus near the cervix. At the knee, a small abscess was discovered just below the inner condyle, occupying the bursæ of the tendons, whence a small quantity of pus escaped, mixed with a glairy fluid. "On passing a probe into the sac, it *might have been* carried into the joint." The cartilages covering the inner condyle had become ulcerated to the extent of a sixpenny piece. The synovial membrane was inflamed.

We agree with Dr. S. that there is no proof, or indeed probability, that this inflammation was of the rheumatic kind. The highly irritable state of the nervous system, connected with the dread of the operation, appears to have rendered the disease fatal under circumstances which, in a better constitution, would not have risked life.

### CHRONIC RHEUMATISM.

We must entirely pass over the dissertation on subacute and chronic rheumatism, with the exception of a page or two, which we shall dedicate to the *treatment* of the latter malady.

General bleeding can hardly ever be necessary or proper in chronic rheumatism.

Sudorifics, in recent cases of the disease, especially when the lumbar and other muscles are affected, are often useful. The combination of volatile tincture of guaiacum, acetate of ammonia, and Dover's powder—or the vinum colchici joined with the two latter medicines, offers a good formula.

Whether as cause or effect, there is often disorder of the digestive organs in chronic rheumatism; and this disorder requires aperients and alteratives. In old and obstinate cases of chronic rheumatism, "a well-conducted mercurial course, so as to produce and keep up a very moderate ptyalism, will sometimes prove successful, after the failure of all other means." The vapour-bath will, of course, prove a powerful auxiliary to the mercurial remedy.

Arsenic, in moderate doses, and not too long continued, has often proved successful.

Bark alone has seldom appeared to our author to exert any anti-rheumatic quality. "But when administered, in confirmed chronic cases, in union either with guaiacum, or with oil of turpentine, I have seen good effects produced." At the Mary-le-bone Dispensary, a mixture of decoction of bark with oil of turpentine is a standard remedy for chronic rheumatism. Unless colchicum produce relief in a few days, Dr. S. does not recommend its continuance.

"A course of sarsaparilla often proves useful in that kind of chronic rheumatism which is accompanied by general derangement of constitution, without the particular affection of any internal organ. We see that, as the health of the system improves, morbid irritability lessens, the flesh of the patient increases, his looks and strength improve, and, gradually, the rheumatic pains pass away. When the periosteum is affected, I have thought it advantageous to give the compound in preference to the simple decoction of sarsaparilla; but, invariably, I direct the use of the cortical part instead of the whole root." 370.

In respect to leeches, cupping, evaporating lotions, blisters, moxas, &c. the practitioner must be guided by the presence or absence of local inflammation in the parts. As a soothing application, our author recommends a plaster containing equal parts of emplastrum opii and cerat. saponis. The warm, vapour, and sulphureous baths are well known remedial agents. The shower-bath, the temperature being gradually lowered to that of the atmosphere at the time, is a powerful remedy. Nor must we forget the magic cures performed by Mahomet, with his baths and shampooing. While Medea's cauldron is boiling at Brighton, what patient would remain encumbered with an old and crazy frame? Let him be dipped and well rubbed, and he is sure to come out a new man! Bandages and Balfour are familiar associations in this complaint.

One hundred pages of the work are filled with *cases* of chronic rheumatism. This department might well have been curtailed—if not omitted in toto. Few medical men, we imagine, will read it.

Two other subjects remain to be noticed before we close this article. These are, neuralgia rheumatica, the principal form of which is sciatica—and neuralgia spasmodica, or tic douloureux. Dr. Scudamore has detailed many cases of sciatica, as illustrating his ideas respecting the treatment of the disease. These we shall pass over. His methodus medendi will be sufficiently appreciated from the following extract.

"When the nervous system is in a state of great irritation, and the pain of the disease excessive, the anodyne treatment is the most desirable to be tried; it being always a point of attention, at the same time, to regulate the digestive functions, and the action of the bowels. If opium in any form should disagree with the patient, conium is usually the most useful substitute.

"Liquor arsenicalis sometimes exerts a specific power over rheumatic neuralgia, but should be given with caution, and not be very long continued.

"Oil of turpentine alone, or joined either with the vinum colchici or the extract from the acetum colchici, sometimes proves successful.

"In one instance, I saw remarkable relief afforded in sciatica by vinum colchici alone; but, for the most part, colchicum has not appeared to be permanently beneficial in this complaint. In the same proportion that sciatica has been associated with error of the biliary system and digestive organs generally; alteratives, aperients, and regulated diet, have produced both palliative and curative effects.

"When the nervous system has been universally in a state of debility, subcarbonate of iron has been useful; but has rarely, in my experience, appeared to possess a specific power in neuralgia rheumatica.

"I have the same observation to make on the properties of the sulphate of quinine.

"The regular use of white mustard seed has, in some instances under my observation, been useful.

"The warm bath may prove useful on general principles of treatment, but does not in a direct manner relieve sciatica.

"A douche of vapor is much more effectual in relieving the affected branches of nerves.

"The Buxton bath is less efficacious in the treatment of sciatica than of the other forms of rheumatism. The shower bath, employed in the advanced stage of convalescence, is a valuable remedy, and in its tonic influence on the whole system exerts a useful control over the affected nerve.

"When there is evident sign of inflammatory action in the nerve or contiguous parts, the abstraction of blood by means of cupping or leeches will be proper. When the affected nerve lies near the surface, leeches are to be preferred. It usually happens in sciatica that, notwithstanding there may be increased vascular action affecting the nerve, it is not of the kind which receives relief from depletion of the blood vessels; and blisters prove more serviceable.

"But in order to obtain decided benefit from the application of blisters, several should be applied in succession, with such frequency as the state of the parts may allow; and this mode I have found more successful than the keeping up ulceration by irritating ointments. When the nerve is affected in different parts of the limb, the situation for the application of the blister should be varied. It sometimes happens that a first or even a second blister does not appear useful, and may even cause an increase of painful irritation; and yet, by perseverance, material benefit is eventually obtained.

"I have not seen any good effects obtained from the employment of tartar emetic ointment in this complaint.

"In one case I saw great benefit produced by rubbing in camphorated mercurial ointment along the course of the affected nerve.

"In acute sciatica, stimulating liniments and friction are hurtful; and, by the converse principle, narcotic applications, as the belladonna fomentation, or plaster, or opium plaster, mentioned in this Treatise, prove soothing and useful.

"In chronic sciatica, and in proportion as there appears to be a deficiency of nervous energy, shewn by remarkable coldness of the limb, and weakness of the muscles, friction and stimulating liniments, oils, or substances in the form of powder,* have a useful influence. When such means fail, a douche of hot water should be tried, and which may be rendered more powerful by impregnation with sulphuretted hydrogen gas, as explained at page 385.

"On the same principle of exciting action, electricity will be proper.

"The support of a roller from the foot upwards usually affords comfort and benefit. In cold weather it should consist of flannel; and, if the nerve be affected near its origin, and especially if lumbago be joined with the sciatica, it is advantageous to extend the roller firmly around the loins." 542.

### NEURALGIA SPASMODICA.

Dr. Scudamore adopts this term from Dr. Kerrison, whose inaugural dissertation, in 1820, was founded on the subject of tic douloureux. We doubt whether "the term neur. spasm. is more descriptive and classical" than the French appellation. Spasm is an involuntary contraction of a *muscle;* whereas the

* "As, for example, a mixture of flour of mustard, salt, and black or even Cayenne pepper."

disease under consideration has no necessary connexion with muscular contraction. Indeed the worst forms of the disease are unaccompanied by spasm, or any other action of the muscles, except what may be occasioned by the pain of the nerve.

Dr. S. bears testimony to the value of carbonate of iron, first introduced by Mr. Hutchinson, though he is obliged to relate cases of its failure. We can hardly expect an universal remedy for any disease, even of the most trifling nature, much less for one like tic douloureux.

Dr. S. as usual, gives the description of the complaint in the form of cases. Of these, we shall only notice one, as being singularly severe and indomitable.

*Case.* A gentleman, aged 55, robust and plethoric—and, before the invasion of this disease, of a most healthy appearance, was subject to occasional attacks of regular gout from the age of 43. He first felt the tic in his right eye, while getting into his carriage, and fancied that the coachman's whip had accidentally touched the eye. He had been three years ill when Dr. S. came to attend him. It was the first case he had ever seen.

"He represented the nature of the pain in these various descriptions—as if stabbed in the eye by a sharp instrument; as if hot needles were suddenly forced into the globe; in the short sleeps which occasional intervals from severe pain allowed at night, he would dream that some one was digging out the eye; and this was the prelude to awaking suddenly in agony.

"Always at the instant of the tic the body was thrown into violent contortion; the breath suspended, with manifest efforts of fortitude and resistance; he experienced the instantaneous sensation of a flash of fire; a suffusion of deep red overspread the cheek and forehead; tears and mucus flowed copiously from the eyes. Membranous ophthalmia was a very troublesome part of the complaint. Sometimes the inflamed and swollen under lid was inverted, sometimes everted. There was great tenderness of the scalp.

"Mastication of any solid food was frequently rendered impossible, from the dread of its being instantly followed by a paroxysm. Even speaking was often avoided for the same reason.

"All the branches of the fifth pair of nerves were affected at different periods; but in and about the eye, the greatest intensity of pain prevailed. The portio dura of the seventh pair was also affected.

"During the day, the interval between the paroxysms seldom exceeded a quarter of an hour; and often was not longer than two minutes. For the most part, the pulse and the natural functions did not deviate materially from a state of health; although occasionally there was great constitutional disturbance, either from the excessive irritation produced by pain or accidental causes, or from the influence of strong medicines tried in the way of experiment." 554.

It is to be lamented that, in this dreadful case, every remedy, including the subcarbonate of iron, completely failed. Several branches of nerves were cut, but to no purpose:—Indeed the disease was rather aggravated, as, on the day after the operation, "the patient complained of a horrid sensation, as if the eye were bursting." About five years from the commencement of these sufferings, this unfortunate gentleman was suddenly released from his miseries by an apoplectic seizure.

It is to be regretted that the dissection was by no means properly made, or sufficiently prosecuted. No examination was made of the state of the affected nerves, or their bony foramina. In the falciform process of the dura mater, at a little distance from the crista galli, a small ossification was found, about a line in thickness, and three in length. The tunica arachnoides slightly adhered to the dura mater, and the pia mater shewed marks of preceding inflammation. There was softening of the brain, and some water in the ventricles.

In the remainder of this section, we find no light attempted to be thrown on the nature or treatment of this terrible malady. We do not wonder at this, since it is evident that we are completely in the dark as to its pathology. Here our analysis must end. We think Dr. Scudamore would have consulted the interest of the profession more, and perhaps his own, if he had omitted a great majority of the cases, and thus compressed the volume into one third its present size. By that plan the work would have had five times the circulation which it will now have, and done much more good. We have given such ample specimens of the work, that our readers will be quite as capable of appreciating its merits as we are. It contains a great body of useful and practical information, though certainly nothing very original or novel in its views of the disease, whether pathological or therapeutical. Still it is the best monograph on the subject of rheumatism which we possess in the English language.

